# Electroosmotic Flow in Mixed Polymer Brush-Grafted Nanochannels

**DOI:** 10.3390/polym8120438

**Published:** 2016-12-16

**Authors:** Qianqian Cao, Hao You

**Affiliations:** 1 College of Mechanical and Electrical Engineering, Jiaxing University, Jiaxing 314001, China; 2 Center for Simulational Physics, Department of Physics and Astronomy, University of Georgia, Athens, GA 30602, USA

**Keywords:** electroosmotic flow, polymer brush, molecular dynamics, dissipative particle dynamics

## Abstract

Mixed polymer brush-grafted nanochannels—where two distinct species of polymers are alternately grafted on the inner surface of nanochannels—are an interesting class of nanostructured hybrid materials. By using a coarse-grained molecular dynamics simulation method, we are able to simulate the electrokinetic transport dynamics of the fluid in such nanochannels as well as the conformational behaviors of the mixed polymer brush. We find that (1) the brush adopts vertically-layered and longitudinally-separated structures due to the coupling of electroosmotic flow (EOF) and applied electric field; (2) the solvent quality affects the brush conformations and the transport properties of the EOF; (3) the EOF flux non-monotonically depends on the grafting density, although the EOF velocity in the central region of the channel monotonically depends on the grafting density.

## 1. Introduction

The implementation of nanochannel functionalities mainly depends on the physicochemical properties of surfaces, which have critical effects on the transport behavior of the confined fluid and the structure of the solvent in the interfacial region [1,2]. A strategy for the modification of the surface properties is to graft polymers with different chemical compositions onto the surfaces of the solid-state channels [3,4]. The grafted polymer layer that creates a soft surface on the nanochannel is also known as polymer brush. The brush has very rich conformational behaviors [5,6,7,8,9,10], and responds to its environment by a change of the chain conformation. Therefore, unlike solid-state channels, the fluid flow in polymer brush-grafted channels is more sensitive to environmental parameters such as the pH, temperature, ionic concentration, light, etc.

In recent years, the electrokinetic transport dynamics of the fluid through nanochannels with soft surfaces modified by polymer brushes permeable to ions and solvent molecules has attracted increasing attention due to a variety of applications [11], such as biomedicine, nanofluidic devices, and molecular filtration. Electroosmotic flow (EOF) is widely used as an important electrokinetic transport mechanism in nanofluidic systems. It is generated when ions within the electrical double layer (EDL) near a charged surface in an electrolyte solution is driven by an external electric field, and can provide a rapid and efficient way to drive or control flows under conditions of extreme confinement. The static and dynamic properties of nanofluidic systems with charged surfaces can stem from the structure of the EDL and the interactions between particles in the EDL and the surfaces. Therefore, understanding the electrohydrodynamics of the fluid in the EDL is critical in many applications, such as species separation, energy storage and conversion, and manipulation of single molecules. To obtain insight into physical mechanisms of electrokinetic transport and EDL structure, numerous computer simulation studies on EOF through the surfaces of solid substrates [12,13,14,15,16,17] have also been reported. However, the EDL structure becomes more complicated in the brush-grafted nanochannel because the properties of the EDL near solid-state surfaces is significantly disturbed by the conformational behavior of the polymer brush. Moreover, due to the coupling between the conformational dynamics of polymer brushes and electrohydrodynamics, it is more difficult to quantitatively predict the transport properties of the electroosmotic flow and the non-equilibrium conformations of the brushes.

Many experimental works have been done to investigate the transport mechanisms of the EOF through polymer-coated channels [18,19,20,21]. Nevertheless, a complete study of the flow properties and the non-equilibrium brush conformations at a molecular scale is still challenging. Based on a scaling law, Harden et al. have studied the coupling characteristics of electrokinetic effects and polymer dynamics at the strong screening limit [22]. Recently, some theories have been established to analyze the electroosmotic transport in a polyelectrolyte-grafted nanochannel with pH-dependent charge density [23] and in a nanocapillary coated with polyelectrolytes [24]. Compared to theoretical analysis, molecular dynamics simulations consider more model details at molecular levels, which is critical for the study of fluid flow in nanochannels; e.g., discontinuity of matter and atomic interactions. Several groups have simulated the modulation of electroosmotic flow in polymer-coated channels using molecular dynamics methods [25,26,27,28,29,30,31,32], providing insights into the role of polymer coatings in controlling the EOF. Additionally, responsive behaviors of charged polymer brushes to the electric field normal to surfaces were also investigated through computer simulations [33,34,35,36].

In this paper, we make the first attempt to explore the electroosmotic transport behavior of the fluid in a mixed polymer brush-grafted nanochannel at the molecular level. The nanochannel is composed of two distinct species of polymers alternately grafted on the inner surface. One is polyelectrolyte chains, and the other is neutral polymer chains. Such nanostructured hybrid materials have recently received growing interest [37], because individual polymer chains exhibit different chain structures and properties upon the application of an external stimulus, and thus might result in special features of the transport dynamics. Recently, Lee. et al. studied macroscopic lateral heterogeneity in mixed polyelectrolyte-neutral polymer brushes [38]. Our results from molecular dynamics simulations indicate that the mixed brush exhibits vertically-layered and longitudinally-separated structures due to the coupling of electroosmotic flow and applied electric field. By introducing various solvent–monomer and monomer–monomer interactions, we are able to model the solvent quality and find that the coating conformations and the coating-induced transport properties of the EOF can be affected by the solvent quality. Particularly, an EOF velocity profile with one step is observed at high grafting density for selective solvents where the polyelectrolyte–solvent contact is favorable. In addition, we find that the EOF flux nonmonotonically depends on the grafting density, although the EOF velocity monotonically depends on the grafting density. These features might open up the possibility of designing functionalized capillary surfaces in nanofluidic systems. The remainder of this article is organized as follows. In the next section, we describe the model system and the simulation method. Following that, the results are presented and discussed. Finally, conclusions are given in Section 4.

## 2. Simulation Details

**Figure 1:**
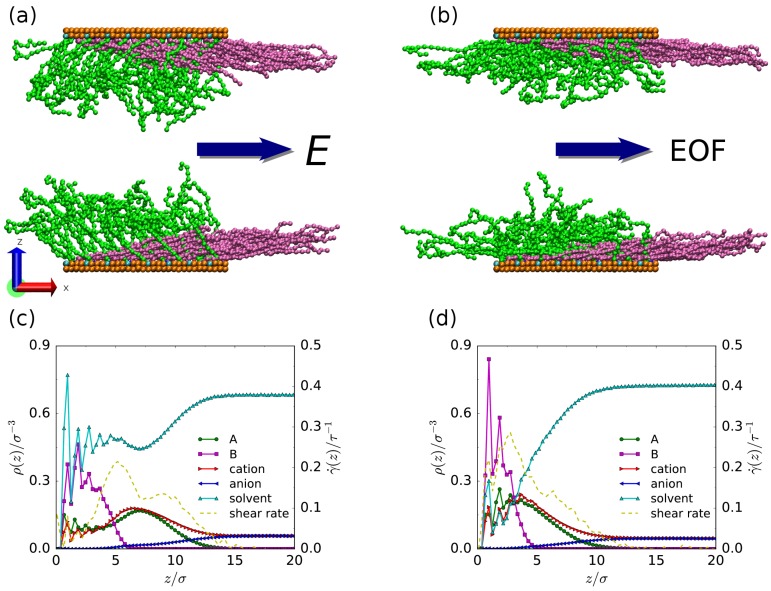
(**a**,**b**) Typical simulation snapshots, and (**c**, **d**) density and shear rate profiles at $\rho_{g}=0.1\sigma^{-2}$. (**a**,**c**) correspond to the B–solvent attraction case; (**b**,**d**) correspond to the A–B attraction case. Cations, anions, and solvent molecules are not shown. Color scheme: negatively-charged A chains (green), neutral B chains (mauve), grafted beads (blue), charged surface particles (cyan), and neutral surface particles (orange). EOF: electroosmotic flow.

We use a coarse-grained model for a system consisting of two flat solid walls coated with mixed polymer brushes and a slab of ionic solution. Fluid particles including ions and solvent molecules are confined between two walls, each of which contains two layers of solid atoms arranged to form a (1 1 1) plane of an FCC crystal. The mixed polymer brush consists of negatively-charged chains and neutral chains, denoted as A and B chains, respectively. Anchored A and B chains are uniformly arranged in a square lattice with the spacing $d=\rho_{g}^{-1/2}$, where the grafting density $\rho_{g}$ denotes the number of end-grafted chains per unit area. To aid in understanding our model, simulation snapshots of the nanochannels coated with polymer brushes are shown in Figure 1.

The intermolecular interactions between different particles include the short-range pair potential, the bond connectivity, and the long-range electrostatic interaction,

(1) $$U=U_{\mathrm{LJ}}+U_{\mathrm{bond}}+U_{\mathrm{ele}}$$

The short-range interaction between any two particles is described by a shifted Lennard–Jones (LJ) potential with the energy scale $\epsilon_{\mathrm{LJ}}$ and the length scale σ,

(2) $$U_{\mathrm{LJ}}\left(r\right)=\left\{\begin{array}{cc} 4\epsilon_{\mathrm{LJ}}\left[{\left(\sigma /r\right)}^{12}-{\left(\sigma /r\right)}^{6}-{\left(\sigma /r_{c}\right)}^{12}+{\left(\sigma /r_{c}\right)}^{6}\right], & r<r_{c}\\ 0, & r\geq r_{c} \end{array}\right.$$

where the cutoff radius for purely repulsive and attractive LJ interactions is set to $r_{c}=2^{1/6}\sigma$ and 2.5σ, respectively. By varying the cutoff radius of different LJ pair interactions [28,39], we simulate six different solvent cases, as follows: (1) pure repulsion between any two components; (2) A–B attraction; (3) A–solvent attraction; (4) B–solvent attraction; (5) A–B/A–solvent attraction; and (6) B–A/B–solvent attraction. Unless otherwise stated, a purely repulsive interaction potential is used with respect to other pair interactions. Interaction energies between different pairs (monomer–monomer, monomer–solvent, and solvent–solvent) are equal to $\epsilon_{\mathrm{LJ}}$. Therefore, excluded volume effects between monomers do not vanish. We only change the cutoff radius to simulate the good solvent (monomer–solvent attraction), poor solvent (monomer–monomer attraction), and athermal solvent (pure repulsion for all pairs) cases. In this work, we do not simulate the theta point, which may be determined by finding the value of monomer–solvent interaction energy. Further details about how to model the quality of theta solvent in molecular dynamics (MD) simulations can be found in Reference [40]. Cut-off radii for different solvent cases are shown in Table 1.

Polymer chains are modelled by a bead–spring polymer model in which all monomers are assumed as coarse-grained beads. The adjacent monomers are connected by a finitely extendable non-linear elastic (FENE) potential with the maximum bond extension $l_{\max}=1.5\sigma$ and the bond strength $k_{b}=30\epsilon_{\mathrm{LJ}}/\sigma^{2}$,

(3) $$U_{\mathrm{bond}}\left(l\right)=-\left(k_{b}l_{\max}^{2}/2\right)\ln\left(1-l^{2}/l_{\max}^{2}\right)$$

The choice of parameters for $l_{\max}$ and $k_{b}$ ensures no unphysical crossing of bonds commonly used in coarse-grained polymer models. The electrostatic interaction between charged particles is described by the Coulomb potential,

(4) $$U_{\mathrm{ele}}\left(r\right)=k_{B}TZ_{i}Z_{j}\frac{\lambda_{B}}{r}$$

where $Z_{i}$ and $Z_{j}$ are the valences of two charged particles. The valence of negatively-charged A monomers is Z=−1, representing a strongly charged brush. We employ the particle–particle/particle–mesh (PPPM) algorithm with an estimated accuracy of $10^{-5}$ to calculate the long-range part of the Coulomb potential [41]. The PPPM algorithm is a well-established method for the calculation of electrostatic interactions. In addition, two different methods have been introduced to solve full electrostatics considering the nature of softly interacting charges in dissipative particle dynamics (DPD) [42,43], with some recent improvements using Ewald sum with vacuum gap boundary conditions due to the confined geometry [17]. The Bjerrum length $\lambda_{B}=e^{2}/\left(4\pi \epsilon_{0}\epsilon_{r}k_{B}T\right)$ that defines the length in the electrolyte over which the electrostatic energy between two unit charges is tantamount to the thermal energy is set to 2σ, where $\epsilon_{0}$ and $\epsilon_{r}$ are the vacuum permittivity and the dielectric constant of the medium, respectively. At room temperature, $\lambda_{B}$ approximates to 0.7 nm for water. Given the system with a slab geometry which is periodic along *x* and *y* directions and possesses a finite length in the *z* dimension, an empty volume with the height of $3L_{z}$ is inserted along the *z* direction to perform three-dimensional simulations with two-dimensional periodicity. Moreover, a correction term is also added to obtain the correct limiting behavior for an infinitely thin slab [44]. An external electric field along the *x*-direction is applied to drive the fluid transport. The external potential is added to each charged particle,

(5) $$U_{\mathrm{ex}}=-Z_{i}eEx_{i}$$

where *E* is the electric field strength and $x_{i}$ is the *x*-directional displacement of the *i*th bead under the external field. The unit of electric field strength *E* is taken as $E^{*}=\epsilon_{\mathrm{LJ}}\sigma^{-1}/{\left(4\pi \epsilon_{0}\sigma \epsilon_{\mathrm{LJ}}\right)}^{1/2}$. If the temperature and the interaction diameter are set to T=300 K and σ=0.3 nm, the corresponding electric field unit is $E^{*}=1.1\;V/\mathrm{nm}$. Here, the electric field is fixed at $E=1.0E^{*}$.

We perform molecular dynamics (MD) simulations under the following conditions: the fluid number density $\rho_{f}=0.81\sigma^{-3}$, the salt concentration $c_{0}=0.025\sigma^{-3}$, the number density of wall particles $\rho_{w}=1.0\sigma^{-3}$, and the surface charge density $\rho_{\mathrm{wc}}=0.1\sigma^{-2}$. The total number of particles in the simulation box remains constant. We found that the normal pressure decreases slightly as the grafting density increases (see Figure S1 in Supplementary Material). Moreover, there is a larger pressure for the pure repulsion case compared to the A–B attraction case due to coalition of A and B chains. All substrate particles are constrained by harmonic restraint potential with spring constant $500\epsilon_{\mathrm{LJ}}/\sigma^{2}$. Each grafted chain consists of $N_{m}=20$ monomers. The dimensions of simulation box ($L_{x}\times L_{y}\times L_{z}$) are 25.4σ×25.4σ×37.3σ. Our MD simulations in an NVT ensemble are carried out by using LAMMPS package [45]. The DPD thermostat [32,46,47] with fraction coefficient $\gamma =1.5\tau^{-1}$ where τ is the time unit is employed to control the temperature at $T=1.2\epsilon_{\mathrm{LJ}}/k_{B}$.

## 3. Results and Discussion

### 3.1. Density Profiles and EOF Velocity

The mixed brush which covers the grafting surface destroys the Debye layer evoked by the surface charges. A new Debye layer builds up near the interface between the brush and the bulk fluid. Thus, the EOF is modulated by the polymer brush. Meanwhile, the EOF through the coating layer can deform the brush in turn, leading to complicated configurations. For visualization purposes, Figure 1a,b shows some typical simulation snapshots of the mixed polymer brush for the A–B attraction and B–solvent attraction cases, respectively. In both cases, neutral B chains are displaced in the direction of the flow (toward the right in the figure) due to the shear force that originates from the electroosmotic transport. They adopt strongly swelling conformations and cling to the grafting surface. Meanwhile, free ends of negatively-charged A chains are longitudinally separated from and vertically located above free ends of neutral B chains, forming a vertically “layered” and longitudinally “separated” structures. A normal electric field can also induce a layered polyelectrolyte brush structure [35]. We can see that the brush conformation is affected by the solvent quality, as follows: (1) The configurations of the polyelectrolyte layer can be quite different. For example, in the B–solvent attraction case, negatively-charged A chains extend into the opposite direction of the flow, resulting from the stronger interaction strength of the electric field in competition with the flow field. In the A–B attraction case, however, some A chains are attracted towards neutral B chains (i.e., the flow direction) due to the enhanced monomer–monomer interaction. (2) Polymer layers in the B–solvent attraction case are thicker than those in the A–B attraction case. This is also confirmed in Figure 1c,d, which presents the density profiles of solvent molecules, monomers, and ions as a function of *z*. Quantitative discussions of the solvent quality effects can be found later.

Comparing the density profiles in Figure 1c,d, more solvent molecules are observed to be squeezed out of the A–B coexistence region for the A–B attraction case. The physical explanation is that introducing the A–B attraction reduces the brush height and leads to a higher monomer density in the vicinity of the grafting surface, in turn inducing a stronger steric repulsion between the monomers and solvent molecules in the region where negatively-charged A chains coexist with neutral B chains. In addition, a large amount of the cations permeate into the mixed brush because of the electrostatic attraction from negatively-charged A chains for both A–B attraction and B–solvent attraction cases.

**Figure 2:**
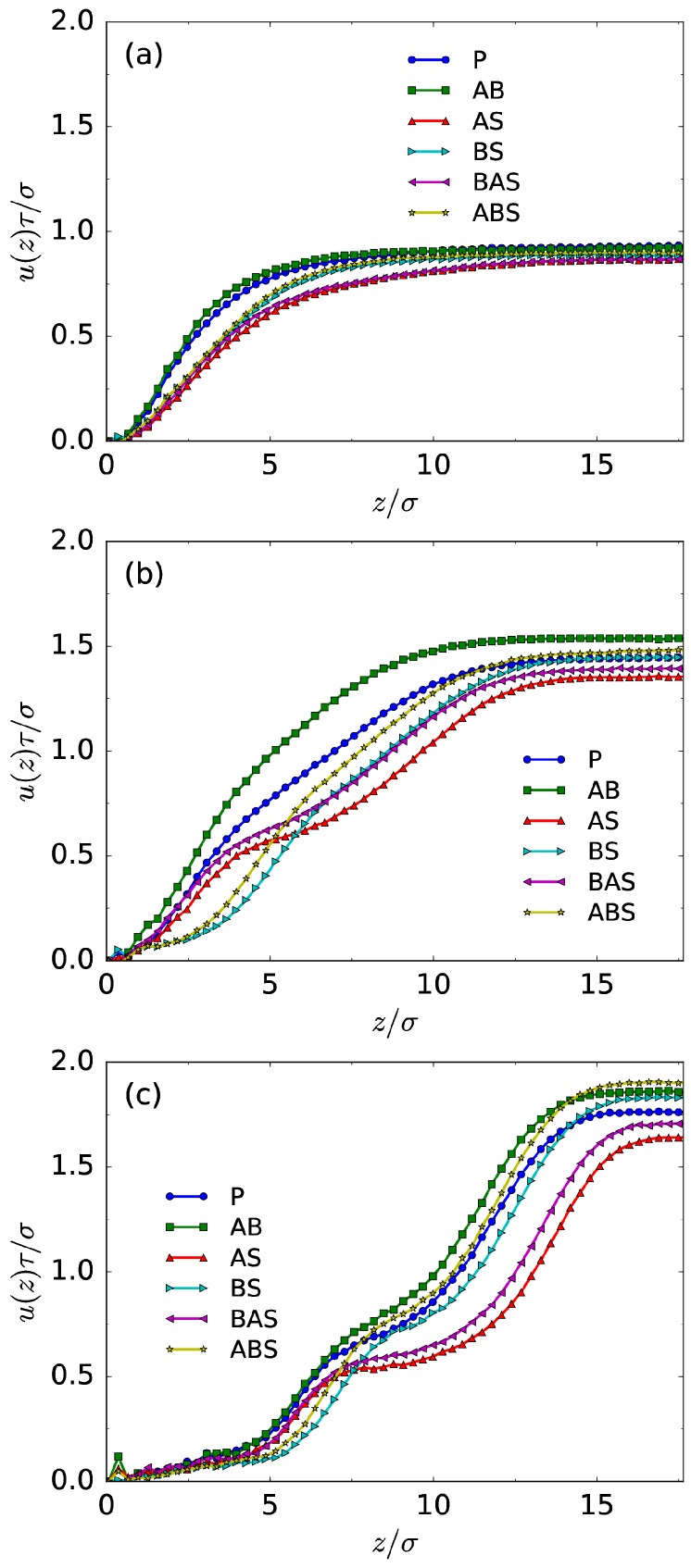
Fluid velocity profiles at grafting densities (**a**) $\rho_{g}=0.02\sigma^{-2}$; (**b**) $0.1\sigma^{-2}$; and (**c**) $0.3\sigma^{-2}$ for different interaction cases. Only the velocity profile to the centerline along the *z* axis is shown. The abbreviations are defined as follows: (1) P corresponds to the case of pure repulsion between any two components; (2) AB represents A–B attraction; (3) AS and BS represent A–solvent and B–solvent attraction, respectively; (4) BAS corresponds the case of A–B and A–solvent attraction; (5) likewise, ABS indicates B–A and B–solvent attraction.

Figure 2 presents the radial variation of the EOF velocity for various LJ interaction types (namely, various solvent qualities) at different grafting densities. We immediately recognize the well-known plug-flow-like character of EOF at a sparse grafting density ($\rho_{g}=0.02\sigma^{-2}$), regardless of the solvent quality. The profiles are quite flat near the channel center, and the shear is located mostly within the polymer–fluid interface from the channel surface. As the grafting density becomes higher, the velocity of bulk fluid through the channel center is enhanced, and the shear occurs in a much wider region. Moreover, the solvent quality effects become more distinguishable at high grafting density. For example, obviously the bulk fluid velocity under the selective solvent condition for polyelectrolytes (such as the A–solvent attraction case) is the smallest shown in Figure 2b,c. The physical reason is that the free edge of the A layer is located above the B layer, introducing favorable contacts with solvent molecules for negatively-charged A chains. Consequently, the A–solvent attraction hinders the motion of the fluid. One particularly interesting transport behavior is the rising EOF profile with one step for the A–solvent attraction and A–B/A–solvent attraction cases in Figure 2c: with increasing values of *z*, the fluid velocity increases slowly in the A–B coexisting area, then steeply in the interface between the B layer and the fluid, slowly again in the A layer, steeply again in the interface between the A layer and the fluid, and finally reaches the steady bulk flow velocity near the channel center. This results from the monomer–solvent interactions in the layered structure of the mixed brush. The friction from the monomers is large in the A–B coexisting layer for solvent molecules, resulting in slow EOF changes near the channel surface. In the B–solvent and A–solvent interfaces, the reduced monomer densities lead to significantly reduced friction forces, and we shall see steep increases occurring in these layers. The A–solvent attraction in the layer where there is only negatively-charged A monomers slows the EOF change for the A–solvent attraction and A–B/A–solvent attraction cases.

Interestingly, for both A–B attraction and B–solvent attraction cases, the shear rate in the interface between the A and B layers (roughly z=5σ) is large and considered as the dominant factor of the stretched configuration of neutral B chains (Figure 1c,d). Furthermore, the shear rate within the B layer (roughly z<5σ) is stronger for the A–B attraction case compared with the B–solvent attraction case, indicating a stronger shear force on the B chains that results in a denser distribution of the B monomers and a reduced brush height (Figure 1d). As for the conformation of negatively-charged A chains, it results from the competition between the electric field and the flow field, and thus can not be understood completely only using the shear rate information.

### 3.2. Transport Properties and Chain Conformation

**Figure 3:**
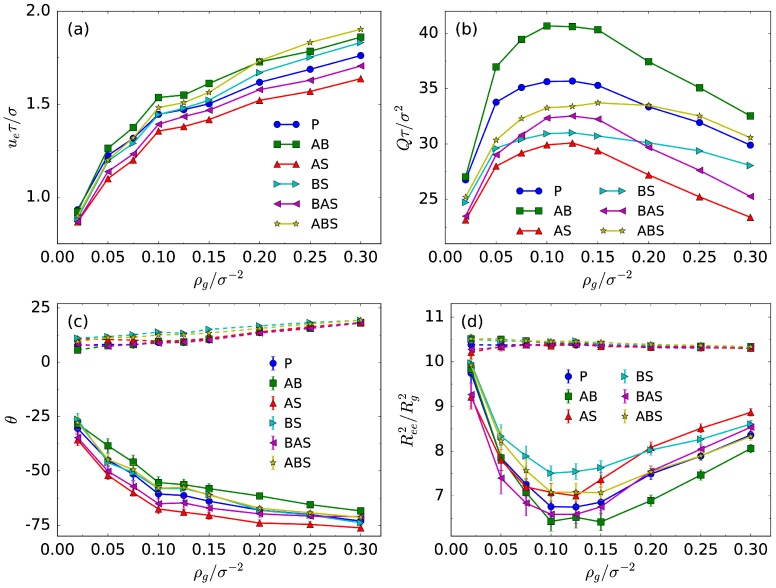
(**a**) Electroosmotic flow velocity $u_{e}$; (**b**) flow flux *Q*; (**c**) inclination angle θ; and (**d**) shape factor $R_{\mathrm{ee}}^{2}/R_{g}^{2}$ as a function of the grafting density. In (**c**,**d**), the solid and dashed lines represent the A and B layers, respectively. The abbreviations (P, AB, AS, BS, BAS, and ABS) have the same meanings as in Figure 2.

To further understand the electrokinetic transport mechanism, the EOF velocity $u_{e}$—defined as the average velocity of particles in the central region of the channel or the plateau value of the velocity profile—and the flow flux *Q* as a function of the grafting density are plotted in Figure 3a,b for various monomer–solvent interactions. As the grafting density increases, $u_{e}$ increases rapidly in the sparse grafting regime and slowly in the dense grafting regime. This hints that in the sparse grafting regime, the increase of the surface charge density due to negatively-charged A chains is an important factor for the fast increase of the EOF velocity. This is different from the role of neutral polymer brushes, which quenches the electroosmotic flow [28,30,32]. In contrast, the increase of the grafting density causes a reduced EOF velocity in the pure repulsion case for neutral polymer-grafted channels [28]. Additionally, the solvent quality effects on the $u_{e}$ are obvious at high grafting densities, which confirms our observation in Figure 2.

From Figure 3b, we find that the EOF flux *Q* non-monotonically depends on the grafting density. With increasing grafting density, $u_{e}$ increases initially, and so does the flux *Q*. However, the maximum of *Q* is observed at a certain grafting density. Further increasing the grafting density leads to increasing $u_{e}$ but decreasing *Q*, due to the reduction in the effective cross-sectional area of the nanochannel. Compared to the bare nanochannel, it is a critical feature for polymer-coated channels. In addition, the magnitude of *Q* is significantly influenced by monomer–solvent interaction types. For example, within all the maximum flux values, the A–B attraction case has a maximum value as large as $40\sigma^{2}/\tau$, while the A–solvent attraction case has a minimum around $28\sigma^{2}/\tau$, which is 30% less.

Figure 3c,d presents more conformational properties of the mixed brush. As can be seen in Figure 1, the mixed brush is deformed with the inclination by the EOF and the electric field. To quantify the tilt degree, we define the average inclination angle as $\theta =\langle \arccos\left(R_{\mathrm{mc}}\cdot s/R_{\mathrm{mc}}\right)\rangle$ for the acute angle, where s is the unit vector along the flow direction and $R_{\mathrm{mc}}$ is the vector pointing to the mass center of the polymer chain from its grafted end. 〈⋯〉 denotes the ensemble average. If θ is obtuse, the inclination angle is set to $\theta -180^{\circ }$. The definition of the orientation angle is well established for description of the orientation of extended polymer chains, as in Reference [31]. In the present cases where the electric field is strong, the inclination of chains occurs mostly in the direction of the electric field, and thus the orientation angle is a good approximation for three-dimensional cases. For a more accurate calculation of the molecular orientation angle, interested readers are referred to Reference [48]. θ as a function of $\rho_{g}$ for negatively-charged A and neutral B chains is shown in Figure 3c. At low grafting densities such as $\rho_{g}=0.02\sigma^{-2}$, θ is smaller than $15^{\circ }$ for neutral B chains. As the grafting density increases, it slightly increases but by no more than $25^{\circ }$, indicating that neutral B chains tilt approximately parallel to the flow direction. Negatively-charged A chains tilt towards the opposite direction of the flow at $\rho_{g}=0.02\sigma^{-2}$ and tend to incline toward the normal to the surface with increasing the grafting density. In Figure 3d, we also compute the shape factor, which is used to describe the overall shape of polymer chains by the definition $S=\langle R_{\mathrm{ee}}^{2}/R_{g}^{2}\rangle$. $R_{\mathrm{ee}}$ and $R_{g}$ are the end-to-end distance and the radius of gyration, respectively. For reference, the shape factors of a free Gaussian chain and a rigid rodlike chain are S=6 and 12, respectively. The larger the shape factor, the straighter the chains. With increasing $\rho_{g}$, the value of *S* for negatively-charged A chains decreases, showing a tendency to collapse. Further increase in the grafting density makes chains more and more swelling. For neutral B chains, however, the shape factor is around 10.5 and changes little as the grafting density changes. This represents a highly-extended conformation of B chains, which is consistent with what we observe in Figure 1.

The conformation transition of the mixed brush results from the coupling among the EOF field, the applied electric field, the LJ potential, and the electrostatic interaction between charged monomers (for negatively-charged A chains only). From the following facts about neutral B chains: (1) they tilt toward the flow direction; (2) and adopt a stretched configuration; (3) further, the solvent quality effect disappears under the dense grafting condition, and we conclude that the shear force due to the EOF dominates the conformational behavior of neutral B chains. As for negatively-charged A chains, the shear force pulls the polymer backbone along the flow direction while the electric field drags it in the opposite direction. In addition, the excluded volume effect and the electrostatic repulsion between the charged monomers cause the A chains to be in an extended state. The resulting configurations are determined by the balance of all these interactions. The applied electric field plays a crucial role in the conformation transition of negatively-charged A chains because θ is always negative, tilting to the direction of the electrostatic force. Meanwhile, it is evident that there is a correlation between the variation of the θ profiles and the EOF velocity $u_{e}$, shown in Figure 3a,c. When $u_{e}$ changes rapidly in the range of $0.02\sigma^{-2}\leq \rho_{g}\leq 0.10\sigma^{-2}$, θ also varies quickly. For $\rho_{g}>0.10\sigma^{-2}$, both $u_{e}$ and θ change slowly. As a comparison, no such correlation can be identified for neutral B chains. This is due to the shearing effect of the EOF field in the layered solution. As we know, negatively-charged A chains are on top of neutral B chains in the layered structure of the mixed brush. Thus, there are more favorable contacts with the solvent for negatively-charged A chains than for neutral B chains. Under the sparse grafting condition, the effect becomes little. When the grafting density increases, the shearing flow gets stronger, and favorable contacts of negatively-charged A chains lead to obvious changes in the A brush configurations. The flow tends to pull negatively-charged A chains along the opposite direction of the electric field until the equilibrium state is reached. We conclude that the electric field dominates the A conformational process under the sparse grafting condition. With increasing grafting density, the coupling between the electric field and the EOF field determines such conformational process. In addition, there are perceptible differences of the A configurations for various solvent qualities. For the cases where there are favorable contacts between neutral B chains and negatively-charged A chains, such as the A–B attraction and B–A/B–solvent attraction cases, the A layer due to the attraction from the B chains contracts towards the B layer, causing an enhanced EOF velocity. Consequently, this feature owing to the coupling between neutral polymer and polyelectrolyte chains does not appear in homogeneous (only neutral polymer or polyelectrolyte) brush systems. For the cases where there are favorable contacts between the solvent and the A chains, such as the A–solvent attraction and A–B/A–solvent attraction cases, some of the A chains are sloped towards the flow direction.

## 4. Conclusions

To summarize, based on molecular dynamics simulations, we first investigate the electrokinetic transport dynamics of a fluid through a channel coated with mixed polymer brush and the conformation transition of the brush. The mixed brush adopts vertically-layered and longitudinally-separated structures where the free ends of negatively-charged polymer (A) chains are vertically located above the free ends of neutral polymer (B) chains, and separated from them in the longitudinal direction. The conformations of neutral B chains are dominated by the EOF field, and that of negatively-charged A chains are determined by the coupling of the applied electric field and the EOF field. Our simulations indicate that the EOF velocity increases with increasing grafting density, while the EOF flux non-monotonically depends on the grafting density with one local maximum. The solvent quality effects are also studied by adjusting various solvent–monomer and monomer–monomer interactions. The conformation of the polyelectrolyte layer in the brush is more apparently influenced by the solvent quality than that of the neutral polymer layer. The solvent quality effects on the transport properties of the EOF become distinguishable at high grafting densities. Particularly, a velocity profile with one step is observed for solutions where the A–solvent contact is favorable. In the present work, we have not studied the conformational transition of mixed polymer brushes and the EOF transport in a non-linear regime originating from the change of applied field strength. We will explore their physical mechanisms in future research.

## Figures and Tables

**Table 1:** Cut-off radii for different solvent cases.

Solvent case	$r_{c}/\sigma$ for A–B	$r_{c}/\sigma$ for A–solvent	$r_{c}/\sigma$ for B–solvent
Pure repulsion	$2^{1/6}$	$2^{1/6}$	$2^{1/6}$
A–B attraction	2.5	$2^{1/6}$	$2^{1/6}$
A–solvent attraction	$2^{1/6}$	2.5	$2^{1/6}$
B–solvent attraction	$2^{1/6}$	$2^{1/6}$	2.5
A–B/A–solvent attraction	2.5	2.5	$2^{1/6}$
B–A/B–solvent attraction	2.5	$2^{1/6}$	2.5

## Supplementary Materials

The following are available online at www.mdpi.com/2073-4360/8/12/438/s1: Supplementary data including a LAMMPS setup script and a figure depicting the normal pressure (Figure S1).
