# Supplementary material for: Electroosmotic Flow in Mixed Polymer Brush-Grafted Nanochannels
Source: Polymers (Basel). 2016 Dec 16;8(12):438. doi: 10.3390/polym8120438 (PMC6431973; doi:10.3390/polym8120438)
Supplement: Supplementary file 1 [file polymers-08-00438-s001.zip › polymers-162294-Suppl.-final.pdf]

Supplementary Materials: Electroosmotic Flow in Mixed Polymer Brush-Grafted Nanochannels

Qianqian Cao and Hao You

The normal pressure as a function of the grafting density for the P and AB cases is shown in Figure S1. Here, we can see that the pressure decreases slightly as the grafting density increases, irrespective of the solvent quality. Due to the assumption in our simulation that the total number of the particles is conserved, there are more polymer and less solvent molecules in the system as the grafting density increases. The bonds in the polymer chains reduce the configuration and mobility entropy contributions to the pressure, resulting in a smaller pressure in the larger grafting density case. Additionally, the pressure for the AB case is smaller than that for the P case. We think it results from the coalition of the A and B chains in the AB case which reduces the system pressure.

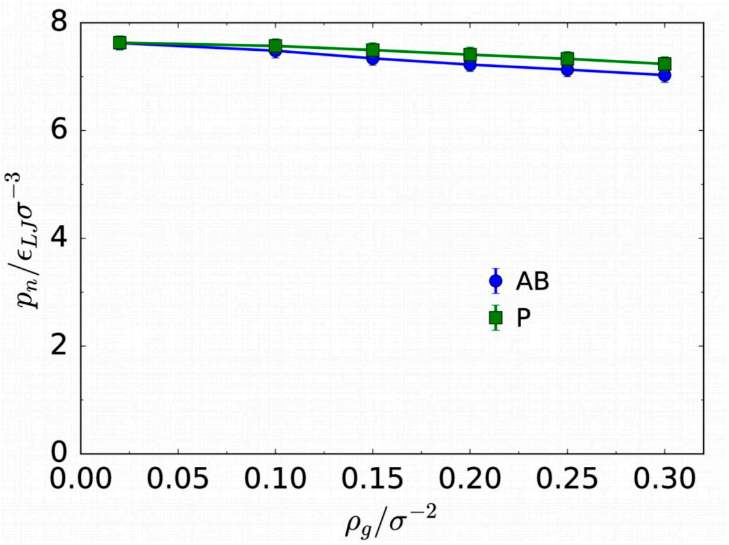

Figure S1. Normal pressure as a function of the grafting density. P corresponds to the case of pure repulsion between any two components, and AB represents AB attraction case.
